# Correlation of N-acetylaspartyl glutamate level in the medial prefrontal cortex with FTND and daily smoking amounts in adult cigarette smokers

**DOI:** 10.3389/fnins.2025.1647427

**Published:** 2025-10-06

**Authors:** Ke Xu, Miaomiao Yu, Liangjie Lin, Man Xu, Jianxin Ren, Qingqing Lv, Mengzhe Zhang, Shaoqiang Han, Weijian Wang, Jingliang Cheng, Yong Zhang

**Affiliations:** ^1^Department of Magnetic Resonance Imaging, The First Affiliated Hospital of Zhengzhou University, Zhengzhou, China; ^2^Key Laboratory for Functional Magnetic Resonance Imaging and Molecular Imaging of Henan Province, Zhengzhou, China; ^3^Engineering Technology Research Center for Detection and Application of Brain Function of Henan Province, Zhengzhou, China; ^4^Engineering Research Center of Medical Imaging Intelligent Diagnosis and Treatment of Henan Province, Zhengzhou, China; ^5^Key Laboratory of Magnetic Resonance and Brain Function of Henan Province, Zhengzhou, China; ^6^Key Laboratory of Brain Function and Cognitive Magnetic Resonance Imaging of Zhengzhou, Zhengzhou, China; ^7^Key Laboratory of Imaging Intelligence Research Medicine of Henan Province, Zhengzhou, China; ^8^Shangcheng County People's Hospital of Henan Province, Xinyang, China; ^9^Clinical and Technical Support, Philips Healthcare, Beijing, China; ^10^Department of Radiology, The Third Affiliated Hospital of Zhengzhou University, Zhengzhou, China

**Keywords:** smoking addiction, neurotransmitter, N-acetylaspartyl glutamate, daily smoking volume, proton magnetic resonance spectroscopy

## Abstract

**Background:**

The neurotransmitter excitation/inhibition (E/I) balance is critical for maintaining normal brain function, and the contribution of nicotine signaling to homeostasis regulation and maintenance of E/I ratios is only beginning to be understood. Advanced *J*-edited ^1^H magnetic resonance spectroscopy (^1^H MRS) enables reliable detection of overlapped brain metabolite, including the neurotransmitters of glutamate (Glu) and N-acetylaspartyl glutamate (NAAG) and gamma-aminobutyric acid (GABA), etc. The purpose of this study was to explore the changes of neurotransmitters in the medial prefrontal cortex (mPFC) of smokers, so as to understand the potential metabolic mechanism of smoking addiction and make a contribution to the cause of smoking cessation.

**Method:**

In 2022, 45 males aged 40–60 years old were recruited. All subjects underwent routine magnetic resonance imaging (MRI) and the *J*-edited ^1^H MRS scans on a 3.0T MRI scanner. The edited spectra were post-processed and quantitatively analyzed using the Gannet tools. Two independent samples *t*-test was used to analyze the differences in GABA, glutamine/glutamic acid (Glx) and NAAG levels between nicotine addicts and control group; Finally, the spearman standard was used to analyze the correlation between metabolite levels and clinical characteristics assessment scales.

**Results:**

All measured metabolite levels in the brain mPFC region of smokers showed no significant difference to those of the control subjects. While the NAAG levels with reference to total creatine or water signals in smokers was significantly correlated with daily smoking volume, and the level of NAAG/Cr was potentially correlated with the FTND score.

**Conclusions:**

In this study, we observed that the level of medial prefrontal NAAG in smokers was associated with daily smoking volume. This suggests that the metabolism of NAAG in the brain is related to nicotine, and the balance of glutaminergic system in the brain of smokers may be disrupted.

## 1 Introduction

The tobacco epidemic has become one of the greatest public health threats facing the world today. Smoke can release thousands of harmful compounds, such as nicotine, tobacco tar, carbon monoxide, and more. Nicotine, found in higher levels in tobacco plants, has long been associated with death and cancer (Chan et al., 2022; Xia et al., 2019; Jun et al., 2024). Studies have shown that the more and the longer you smoke, the risk of various diseases is significantly increased (Chan et al., 2022). Even though smoking is known to be harmful to health, most people find it difficult to quit after smoking. The reason could be complicated. On the one hand, tobacco is addictive; on the other hand, the body will appear a series of unbearable symptoms during the process of quitting smoking to force relapse, such as insomnia (Jaehne et al., 2009), irritability, mood swings (Shiffman, 2008), depression (Langdon et al., 2013), and physical discomfort, etc. collectively referred as withdrawal syndrome (Smith et al., 2014). For smokers who have difficulty quitting smoking, accurate treatments and interventions have not yet been found. In addition, given the harmful effects of smoking on the brain (McClernon et al., 2016; Chang et al., 2024), understanding the underlying neurobiological addiction mechanisms of smokers is important to identify effective approaches to tobacco withdrawal.

Excitation/inhibition (E/I) balance of neurotransmitters is vital for maintaining normal brain functions (Jessen et al., 2013), while the contributions of nicotinic signaling to homeostatic regulation and maintenance of the E/I ratio are only beginning to be understood. Nicotine, the main harmful substance in tobacco, binds to receptors after entering the body, desensitizing and upregulating nicotinic acetylcholine receptors, resulting in the release of neurotransmitters in the central nervous system (Yizhar et al., 2011). Nicotine addiction is caused by neural adaptation of a complex and extensive set of brain regions and neurotransmitter systems. Previous studies have suggested that nicotine addiction mainly involves the dopaminergic neurotransmitter system (Yizhar et al., 2011), but other neurotransmitters are increasingly mentioned, including the glutaminergic and gamma-aminobutyric ergic systems.

Proton magnetic resonance spectroscopy (^1^H MRS; Graveron-Demilly, 2013) provides a non-invasive method for studying the changes of brain metabolism in smokers. The main brain metabolites including N-acetyl-aspartic acid (NAA), creatine (Cr), choline (Cho), glutamic acid (Glu), and myo-inositol (mI), etc. can be reliably detected by the conventional MRS techniques [e.g., Point RESolved Spectroscopy (PRESS)]. While for specific measurements of some brain metabolites with relatively low concentration (usually overlapped), such as the inhibitory neurotransmitter of gamma-aminobutyric acid (GABA), and the excitative neurotransmitters of N-acetylaspartyl glutamate (NAAG) and aspartate (Asp), the advanced *J*-edited ^1^H MRS technique (MEscher-GArwood Point RESolved Spectroscopy, MEGA-PRESS) was recently developed (Harris et al., 2017). Since the chemical shifts and *J*-coupling constants are commonly different among metabolites, the MEGA-PRESS sequence was usually seperatively optimized. At present, MRS's research on nicotine addiction mainly focuses on areas of interest such as the anterior cingulate cortex (a key region of the reward circuit; Janes et al., 2016; O'Neill et al., 2023), the prefrontal cortex (Durazzo et al., 2016; Yu et al., 2023; Bagga et al., 2018), and the hippocampus (Gallinat et al., 2007). The mainly interested metabolites are NAA (O'Neill et al., 2023; Gallinat et al., 2007), Cr (O'Neill et al., 2023), glutamine/glutamic acid (Glx; Janes et al., 2016; Li et al., 2018), aspartate (Asp; Yu et al., 2023), GABA (Durazzo and Meyerhoff, 2021; Schulte et al., 2017) and so on. However, the results of the current studies are not consistent, and more relevant studies are needed in the future.

Structural MRI (sMRI) studies have shown that smoking causes morphological changes of brain (Yang et al., 2020; Li et al., 2015; Fritz et al., 2014; Zhong et al., 2016); and functional magnetic resonance imaging (fMRI) studies have shown that chronic smoking alters the activity of regions in the brain involved in attention, working memory processes, high-level cognitive processing, anxiety, and reward processing (Musso et al., 2007; Weng et al., 2021; Lin et al., 2020). The medial prefrontal cortex (mPFC) is crucial for cognition, emotion regulation, and executive control (Kringelbach, 2005; Kalivas and Volkow, 2005), and is also a key area in models of brain circuits related to substance addiction. From the perspective of structural and functional MRI studies, the mPFC region has also been considered as one of the most abnormal brain regions involved in the formation of nicotine addiction behavior. At the same time, some studies (Durazzo et al., 2016; Bagga et al., 2021) have shown that metabolites in the prefrontal lobes of smokers have changed, which may be related to impaired cognitive function and addiction. Therefore, in this study, we focuses on changes of brain metabolism in nicotine addicts in the medial prefrontal cortex region.

Previously, our team found that compared with the control group, Asp levels were increased in the mPFC of the smoking group, and nicotine may be an important cause for the change of Asp levels in the brain (Yu et al., 2023). It is worth to explore whether the other neurotransmitters (such as GABA, Glu and NAAG) also change in smokers, and how is their relationship with nicotine intake. We hypothesized that levels of GABA, Glx, and NAAG in the medial prefrontal lobe of the brain may also be related to nicotine intake in nicotine addicts (NA). In this study, the MEGA-PRESS technique was used to assess metabolite levels in smokers and non-smokers, and the relationship between brain metabolite levels and clinical scales of smoking was evaluated.

## 2 Materials and method

### 2.1 Study subjects

From March to August 2022, a total of 45 male volunteers (between 40 and 60 years old) were recruited in the Henan Province of China through the promotion of the wechat platform, including 21 smokers and 24 non-smokers. Since female smokers are relatively rare and difficult to recruit, only male participants are currently enrolled in this study. In the smoker's group, all subjects smoked no less than 10 cigarettes per day, smoked for more than 2 years. The fagerstrom Nicotine Dependence Scale (FTND) was used to evaluate nicotine dependence. And in our study, we ensured that the control group participants had no history of smoking, and both groups were matched in terms of age, gender, and educational level. We excluded volunteers with underlying diseases or mental disorders, intracranial tumors or major head trauma, substance dependence or behavioral dependence history, recent medication usage within the past month, or contraindications for MRI scans.

All volunteers received a set of questionnaires, include basic information (age, sex, height, weight, etc.), FTND scale, Russell Reason for smoking questionnaire (RRSQ), and Barratt impulse Scale (BIS-11). Eligible participants were told about the purpose of the study, safety instructions, and what to expect during the scan, and then began the MRI scan. The questionnaire was conducted in Chinese and all participants were Chinese native speakers. Each participant signs an informed consent form after knowing all the necessary information. This study was approved by the local Medical Ethics Committee of the First Affiliated Hospital of Zhengzhou University.

### 2.2 MRI examination equipment and sequence parameters

**Figure 1:**
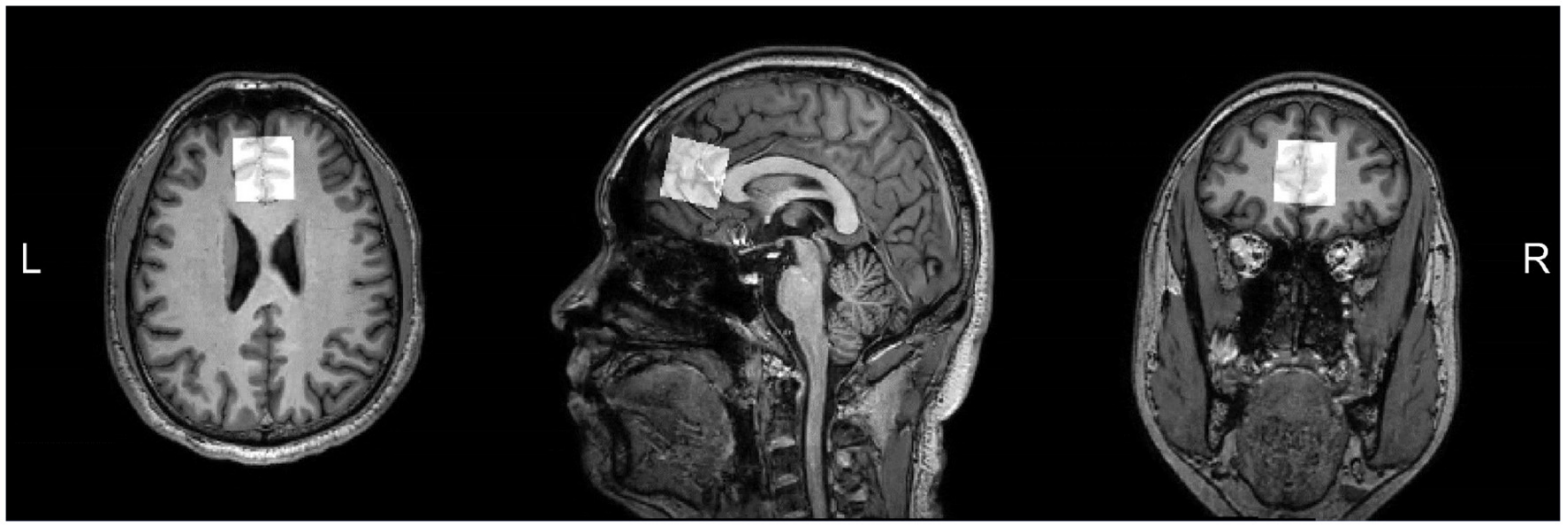
T1-weighted images showing MRS voxel placed in the mPFC on axial, sagittal, and coronal sections.

All subjects were scanned on a 3.0T MRI scanner (Ingenia CX, Philips Healthcare, Best, The Netherlands) equipped with a 32 channel heal coil in the Magnetic Resonance Department of the First Affiliated Hospital of Zhengzhou University. During the scan, subjects should stay awake and not fall asleep, and spongy pads were placed near their ears on both sides to stabilize their heads. In order to eliminate the interference of other organic brain lesions, we first collected some routine sequences for clinical diagnosis: including T_1_ weighted imaging (T_1_WI), T_2_ weighted imaging (T_2_WI), fluid-attenuated inversion recovery (FLAIR), and diffusion weighted imaging (DWI). For anatomical reference, a high-resolution 3D T_1_WI scan were carried out using the gradient echo sequence with following parameters: number of sagittal slices = 200, field of view (FOV) = 256 mm^2^, repetition time (TR) = 9 ms, echo time (TE) = 4 ms, slice thickness = 0.9 mm, and acquisition time = 3 min 26 s. The MEGA-PRESS sequence for GABA and Glx spectral editing was implemented with parameters as follows: TR = 2,000 ms; TE = 68 ms; number of signal averages (NSA) = 192 (both ON and OFF spectra were repeated by 96 times, totally 192 spectra); ON/OFF frequencies = 1.9/7.5 ppm; scan time = 6 min 30 s. The ON and OFF experiments of the MEGA-editing sequence were performed in an interleaved Fashion, and the VAPOR scheme was selected for water suppression (Tkáč et al., 1999). The acquisition of *J*-edited NAAG spectrum is also carried out using MEGA-PRESS sequence, and the parameters (Edden et al., 2007) was implemented as follows: TR = 2,000 ms; TE = 140 ms; NSA = 192; ON/OFF frequencies = 4.61/4.15 ppm; scan time = 6 min 30 s. The bandwidth of the editing pulses for both GABA/Glx and NAAG editing was 100 Hz. Unsuppressed water signals were recorded with 32 signal averages as an internal concentration reference. T_1_-weighted MPRAGE structure image provided reference for VOI localization in spectral scanning experiments, and the spectral data were obtained in a volume of 30 × 30 × 30 mm^3^ located in the frontal lobe of the brain (shown in Figure 1). FASTMAP shimming of the voxels was performed automatically before each acquisition, yielding water signal line widths of 6 to 10 Hz.

### 2.3 Data processing

**Figure 2:**
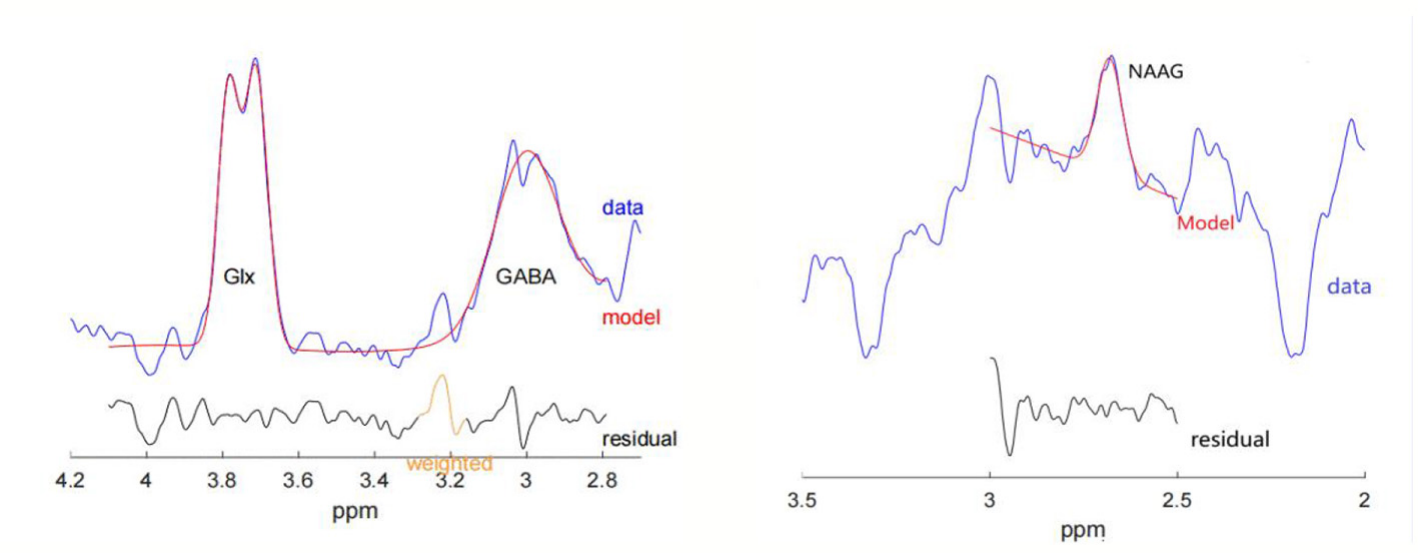
Examples of the acquired *J*-edited spectra and fitting results. The red line represents the standard curve of the fitting model, the blue line represents the target metabolite profile of the edited data, and the black line represents the residual between the standard curve and the actual metabolite profile obtained.

The Gannet 3.0 software based on MATLAB (http://www.gabamrs.com/) was used to spectral post-processing and quantification (Edden et al., 2014). The GABA signal detected by MEGA-PRESS might contain co-edited signal from macromolecules and homocarnosine, so it is referred as GABA+ below. First, the spectral registration module developed by Near et al. (2015) was used for frequency and phase correction on each of the acquired ON or OFF spectrum (aligned to “SpecReg” for GABA+ edited spectra, and aligned to “Cr” for NAAG edited spectra), especially for removing subtractive artifacts associated with subject motion and scanner drift, so as to improve the data quality. Then the *J*-edited spectrum was generated by averaging the ON and OFF spectra, respectively, and then substracting the OFF-averaged spectrum from the ON-averaged spectrum. The line broadening of 3 Hz was used for each edited spectrum. Second, for GABA+ editing, the edited GABA+ signal at 3.02 ppm was fitted using a single Gaussian model, while the Glx signal at 3.75 ppm was fitted using a double Gaussian model. For the NAAG editing, the edited NAAG signal at 2.60 ppm was fitted using a single Gaussian model. The tCr signal at 3.0 ppm from the OFF-averaged spectrum and the extra acquired 32-averaged water signal were fitted with a single Lorentz model and a mixed Lorentz-Gaussian Model, respectively, for references. Gannet initially obtained the sub-peak area of metabolites such as GABA+, Glx, NAAG, tCr and water signals, as well as the full width at half maximum (FWHM) and fitting errors (FitErr). The fitting error, that is, the deviation of the fitting peak amplitude between the used model and the actual spectrum in the fitting process. According to the FWHM and FitErr of metabolites, the overall quality and the smoothing effect of spectral data were judged. The spectral fitting curve obtained is shown in Figure 2. The SPM12 (https://www.fil.ion.ucl.ac.uk/spm/software/download/) was used to align the acquired MRS voxel to a standard brain image and segment the 3D T1WI image into gray matter (GM), white matter (WM), and cerebrospinal fluid (CSF), recording the composition ratio information of the three components within the MRS voxel; Finally, the levels of GABA+, Glx, NAAG on the scale of water signal corrected by CSF tissue were estimated from the metabolite peak amplitude. In order to observe whether there is any change in tCr and other main brain metabolites, the OFF-averaged spectra for GABA editing were analyzed using Tarquin software (https://tarquin.sourceforge.net/) for quantification of metabolites, including tCr, tNAA, tCho, and mI (Wilson et al., 2011).

### 2.4 Data analysis (statistical analysis)

To ensure the quality of the spectrum data, metabolite levels were included in further statistical analysis only when the fitting error limits of GABA+/Cr and Glx/Cr were less than 15%, but NAAG, due to its relatively low concentration, used data with fitting error limits of less than 25%. The Example Fits for Worst-Case Scenarios can be found in the Supplementary Figure 1.

The SPSS 22.0 software was used for statistical analysis of clinical data of smoker and HC groups in our study. Normal test was conducted using the Kolmogorov-Smirnov test before analysis; If the variables meet the normal distribution criteria, the independent sample *t*-test is used, otherwise the non-parametric test is used. The clinical characteristic variables (age, height, weight, education level, daily smoking amount, initial of smoking age, FTND score, BIS-11 score, and RRSQ score) of smoker and HC groups were compared, and the measurement data were expressed as mean ± standard deviation (X ± SD). The levels of metabolites (GABA+, Glx, NAAG (Refer to water or tCr), tCr, tNAA, tCho, and mI) in the mPFC between the smoking group and the control group were also compared, and *p* < 0.05 was considered statistically significant. And then, the correlation between GABA+, Glx, NAAG levels (Refer to water or tCr) and clinical characteristics assessment scales (including daily smoking volume, smoking age, FTND score, RRSQ score, and BIS-11 score) was analyzed by Spearman criteria. Finally, multiple comparisons were corrected using false discovery rate (FDR) control at 0.05 within each group of correlations.

## 3 Results

### 3.1 Clinical and demographic information

Three subjects (2 smoker and 1 non-smoker subjects), of which two with brain-based diseases and one with alcohol dependence, were excluded. Subjects with poor data quality were also excluded (including 3 smoker and 1 non-smoker subjects in GABA+ and Glx level analyses; 3 smoker and 5 non-smoker subjects in NAAG level analyses). The GABA+ and Glx levels were finally analyzed in 16 smokers and 22 age- and sex-matched non-smokers. The NAAG levels were analyzed in 16 smokers and 18 age- and sex-matched non-smokers. The demographic characteristics of the control group and the smoking population are presented in Table 1. Results indicated no significant differences in age, height, weight, or years of education between the two groups. The FTND scores revealed that the smokers included in this study had moderate nicotine dependency levels (mean FTND scores > 4.0).

**Table 1:** Clinical and demographic data.

**Characteristics**	**GABA**+ **and Glx analyses**			**NAAG analyses**		
	**Smokers (*n* = 16)**	**Non-smokers (*n* = 22)**	***P* value**	**Smokers (*n* = 16)**	**Non-smokers (*n* = 18)**	***P* value**
Age (years)	48.31 ± 6.58	47.77 ± 6.39	0.801	49.75 ± 6.39	47.83 ± 6.13	0.379
Height (cm)	170.62 ± 4.19	171.73 ± 5.83	0.524	171.5 ± 4.27	172.00 ± 6.37	0.793
Weight (kg)	69.50 ± 12.49	74.27 ± 9.06	0.180	70.87 ± 12.23	76.22 ± 6.96	0.251
Years of education	10.62 ± 3.22	12.23 ± 4.45	0.229	11.00 ± 3.37	11.83 ± 4.76	0.564
Initial age of smoking	19.87 ± 3.59	–	–	19.25 ± 3.23	–	–
Years of nicotine use	28.00 ± 6.28	–	–	30.06 ± 6.55	–	–
Daily smoking amounts	17.56 ± 7.54	–	–	18.19 ± 8.15	–	–
**Clinical questionnaires**						
FTND score	4.50 ± 2.42	–	–	4.25 ± 2.46	–	–
RRSQ score	23.69 ± 14.11	–	–	22.69 ± 12.92	–	–
BIS-11 score	55.00 ± 6.45	–	–	55.31 ± 7.98	–	–

### 3.2 Comparison of metabolite levels between the two groups

The spectra for quantification of GABA+, Glx, and NAAG levels by *J*-edited technology were successfully obtained from the mPFC brain region of included subjects. The GABA+, Glx, and NAAG levels (refer to water or tCr signals), as well as the tCr, tNAA, tCho, and mI levels showed no significant difference between the two groups (Table 2). The FWHM, FitErrs of metabolites, tissue compositions, and the absolute frequency offset also showed no significant difference between groups. The statistical power along with detectable effect sizes for the metabolite comparison between smokers and controls (Supplementary Table 3). The differences of NAAG signals between smokers and controls (either with reference to Cr or water signal) were close to the detectable effect sizes (0.007 vs. 0.010, and 0.091 vs. 0.120).

**Table 2:** Relative concentrations of GABA+, Glx, and NAAG in the medial prefrontal cortex was compared between the Smoking group and HC group.

	**Smokers (*n* = 16)**	**Non-smokers (*n* = 22)**	***P* value**
GABA+/Cr	0.097 ± 0.046	0.091 ± 0.009	0.550
Glx/Cr	0.114 ± 0.104	0.131 ± 0.172	0.733
GABA+/water (i.u.)	2.498 ± 0.507	2.604 ± 0.276	0.411
Glx/water (i.u.)	9.732 ± 1.966	9.783 ± 0.952	0.916
Fraction_GM_	0.491 ± 0.051	0.519 ± 0.039	0.072
Fraction_WM_	0.345 ± 0.050	0.320 ± 0.038	0.093
Fraction_CSF_	0.163 ± 0.030	0.161 ± 0.038	0.821
FWHM_H2O_ (HZ)	10.781 ± 2.444	10.459 ± 1.468	0.616
FitErr_GABA+_ (%)	7.537 ± 2.831	7.486 ± 2.185	0.950
FitErr_Glx_ (%)	4.825 ± 1.889	4.954 ± 1.374	0.808
tNAA (mM)	3.798 ± 0.762	3.612 ± 0.509	0.371
tCr (mM)	7.694 ± 0.756	7.622 ± 0.873	0.793
tCho (mM)	1.846 ± 0.391	1.866 ± 0.279	0.850
mI (mM)	5.371 ± 0.795	5.293 ± 0.842	0.775
Absolute frequency offsets (F0, ppm)	4.691 ± 0.046	4.706 ± 0.048	0.354
	**Smokers (*****n*** = **16)**	**Non-smokers (*****n*** = **18)**	***P*** **value**
NAAG/Cr	0.038 ± 0.014	0.031 ± 0.010	0.063
NAAG/water (i.u.)	0.470 ± 0.166	0.379 ± 0.122	0.086
FWHM_H2O_ (HZ)	8.912 ± 1.476	8.983 ± 1.232	0.880
FitErr_NAAG_ (%)	17.569 ± 3.905	18.206 ± 3.391	0.614
Fraction_GM_	0.490 ± 0.050	0.514 ± 0.032	0.112
Fraction_WM_	0.346 ± 0.041	0.324 ± 0.035	0.103
Fraction_CSF_	0.164 ± 0.031	0.162 ± 0.039	0.915
Absolute frequency offsets (F0, ppm)	4.680 ± 0.027	4.673 ± 0.054	0.667

### 3.3 Correlations of brain metabolite levels with clinical scales of smoking

**Figure 3:**
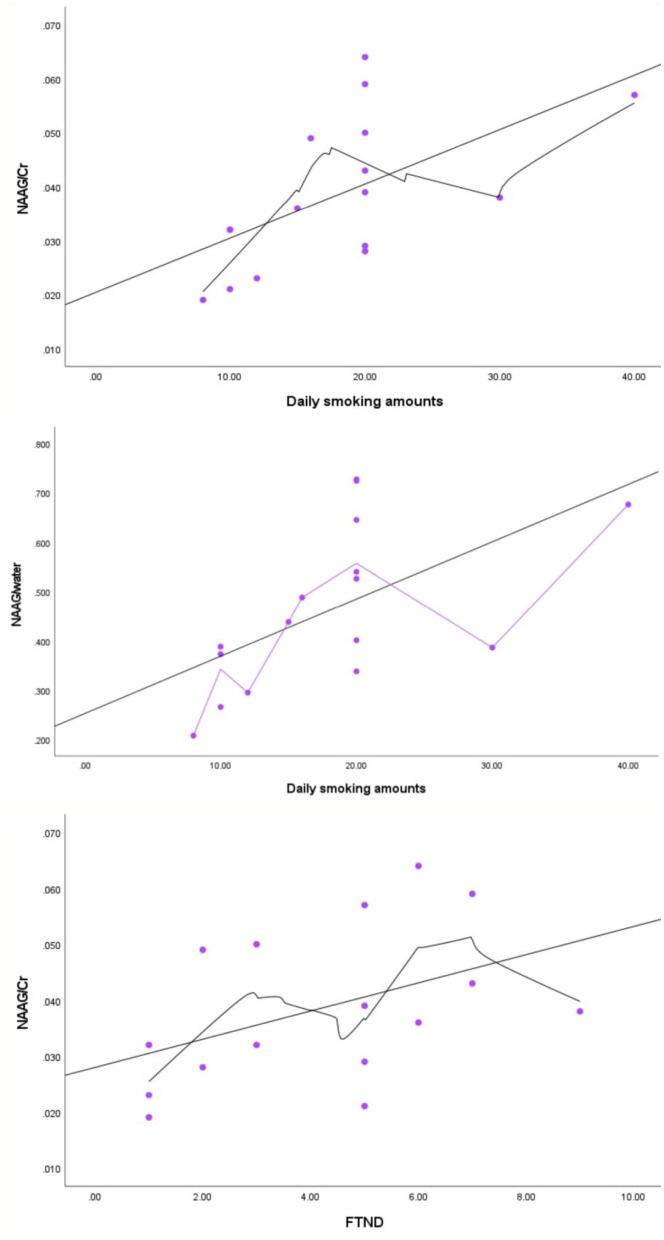
Correlations of the NAAG level with the daily smoking amounts and the FTND score in smokers.

The NAAG/Cr level was positively correlated with the FTND score (*r* = 0.535, *p* = 0.033) and daily smoking amounts (*r* = 0.647, *p* = 0.007). Meanwhile, the NAAG/water level was positively correlated with daily smoking amounts (*r* = 0.644, *p* = 0.007). However, no significant correlation was found between NAAG (Refer to water or tCr) levels and the years of nicotine use, the initial ages of smoking, the BIS-11 score or the RRSQ score. No significant correlation was also found between GABA+ or Glx (Refer to water or tCr) levels and the clinical scales (see Table 3 and Figure 3). The NAAG/Cr (*p* = 0.042) and NAAG/water (*p* = 0.042) levels were still positively correlated with the daily smoking amount even after FDR correction (Supplementary Table 2). While the correlation between NAAG/Cr and the FTND score became unsignificant after correction (*p* = 0.099).

**Table 3:** Spearman correlation analysis between neurotransmitter levels and smoking assessment scales.

	**GABA**+**/Cr**		**Glx/Cr**		**NAAG/Cr**	
	***r* value**	***P* value**	***r* value**	***P* value**	***r* value**	***P* value**
FTND	0.276	0.301	0.149	0.581	0.535	0.033^*^
RRSQ	0.297	0.265	0.365	0.164	0.437	0.090
BIS-11	−0.061	0.824	0.067	0.806	−0.075	0.783
Initial age of smoking	−0.123	0.649	0.197	0.463	−0.456	0.076
Daily smoking amounts	0.489	0.055	0.119	0.660	0.647	0.007^*^
Years of nicotine use	−0.359	0.179	−0.451	0.080	0.215	0.425
	**GABA**+**/water**		**Glx/water**		**NAAG/water**	
	***r*** **value**	***P*** **value**	***r*** **value**	***P*** **value**	***r*** **value**	***P*** **value**
FTND	0.213	0.429	0.054	0.843	0.434	0.093
RRSQ	0.289	0.278	0.021	0.939	0.509	0.086
BIS-11	−0.216	0.422	0.126	0.643	−0.037	0.892
Initial age of smoking	0.246	0.358	−0.110	0.685	−0.416	0.109
Daily smoking amounts	0.170	0.530	−0.109	0.688	0.644	0.007^*^
Years of nicotine use	−0.201	0.456	0.028	0.918	0.286	0.283

## 4 Discussion and conclusion

In this study, we successfully detected brain GABA+, Glx, and NAAG levels (Refer to water or tCr) of smokers and control subjects in the mPFC region using *J*-editing spectroscopy. Results show that there is no significant difference in metabolite levels in mPFC of the brain between smokers and non-smokers. However, we discovered that the medial prefrontal NAAG level (refer to tCr or water) in smokers was significantly correlated with daily smoking volume, and the potential correlation of NAAG/Cr level with the FTND score.

No significant change was detected in GABA+, Glx, and NAAG levels in the brain mPFC in smokers compared to non-smokers, which may be related to that the subjects in our study were men with moderate nicotine tolerance and the sample size was relatively small. The mPFC of male smokers does not seem to be a region where these brain metabolites are affected, or the differences are too small. These findings align with the research conducted by Bagga et al. (2018), which reported no significant difference in GABA levels between male smokers and non-smokers in the prefrontal cortex; however, female smokers exhibited markedly elevated GABA levels compared to their non-smoking counterparts. Another investigation (Schulte et al., 2017) also revealed no substantial differences in GABA concentrations among smokers, smokers using multiple substances, and healthy controls. However, a recent study on chronic smoking by Durazzo and Meyerhoff (2021) found that the level of GABA in the right dorsolateral prefrontal cortex of smokers (of whom 89% were male) was significantly lower than that of non-smokers, but the GABA levels in the anterior cingulate cortex were similar in both groups; and it proved that the concentration of GABA in the anterior cingulate cortex and the dorsolateral prefrontal cortex was related to the neurocognitive and decision-making/impulsive behaviors of smokers. And Epperson et al. (2005) observed that female smokers had lower GABA levels in the occipital cortex than female healthy controls. Consequently, Shyu et al. (2022) propose that regulation of GABA level is related to smoking, and nicotine dependence may influence GABA concentration in a manner that is both sex-dependent and brain region-specific.

In contrast to our findings, (Bagga et al. 2021) observed that Glx/Cr and Cho/Cr ratios in the prefrontal brain region were lower in smokers than in non-smokers; However, these changes only emerged when they considered the interactions between the groups and NAAG/Cr. Therefore, their study highlights that it is not one single metabolite, but the interactions of multiple metabolites, that causes the significant differences in brain metabolism between smokers and non-smokers and under different task conditions. (Schulte et al. 2017) found that there were differences in the absolute concentration of Glx in the dorsal anterior cingulate cortex between smokers and healthy controls; and both the smokers and multiple substance users had higher Glx levels compared to healthy controls. Since there was no difference in nicotine dependence between smokers and multiple substance users, this may indicate that changes in Glx concentration may be more associated with smoking dependence. (Mashhoon et al. 2011) used ^1^H-MRS to detect metabolites in the brain of smokers and found that compared with people who were able to persist in quitting their addiction with the help of Nicotine Replacement Therapy (NRT), the Glu/Cr level in the dorsolateral anterior cingulate cortex was significantly decreased in those who could not quit smoking (the relapse-prone group). The study of (Mennecke et al. 2014) showed that the concentrations of Glx and Cho in the left cingulate cortex of smokers were significantly higher than those of non-smokers, and 6 out of 7 smokers returned to normal Glx concentration 3 days after quitting smoking, but no changes were observed in Cho, proving that the physiological metabolism of human brain is related to nicotine addiction and withdrawal. (Gutzeit et al. 2013) quantitatively detected metabolites in the insular cortex of smokers and non-smokers found that during withdrawal, the Gln level in the insular cortex of smokers was significantly increased, and the Glu level was slightly increased. A study by (O'Neill et al. 2014) found no significant difference between smokers and non-smokers in Glu levels in the thalamus (a brain region rich in nicotinamide acetylcholine receptors), but among smokers, the number of cigarettes smoked per day and the number of years smoked were strongly associated with reduced Glu levels in the thalamus. Therefore, considering that the selection criteria of research objects are slightly different and there are many factors affecting MRS spectral quality, the results of various studies cannot reach a completely consistent conclusion. In general, however, the balance of glutaminergic systems in the brain of smokers is somewhat disrupted, and the specific situation needs to be studied more accurately.

Although there was no significant difference in metabolite levels between the two groups, this study found that the level of NAAG/Cr and NAAG/water in the mPFC of smokers was significantly correlated with the daily smoking amount even after the FDR correction. These results suggest that the metabolism of NAAG in the mPFC of the brain is related to nicotine intake and may play an important role in the development of nicotine dependence. The level of NAAG/Cr was also observed to be positively correlated with the FTND score, while the correlation did not reach the significance threshold after FDR correction (*p* = 0.099). This result indicates that the initial significant outcome might be partly attributed to the contingency of multiple tests and failed to pass stricter control over the error detection rate, and there is a potential correlation between NAAG/Cr and FTND. In the future, these potential associations need to be further verified through expanded samples or targeted experiments to prove this.

The neurotransmitter NAAG is a selective endogenous agonist of metabotropic glutamate receptor 3 (mGluR3), which can regulate the release of glutamate; and is also the third-most-prevalent excitatory neurotransmitter in the human nervous system. NAAG is formed from NAA and glutamate, and it may play import roles in neuro disorders such as traumatic brain injury and amyotrophic lateral sclerosis (Neale et al., 2005). The imbalance of glutamatergic neurotransmission was thought to be one of the central mechanisms contributing to psychiatric diseases. Recent research (Li et al., 2014) also suggests the glutamatergic system as a promising target for pharmacological interventions, with inhibition of mGluR1 and mGluR5, or stimulation of mGluR2/3 preventing priming- and cue induced reinstatement of nicotine seeking in preclinical models. One study showed that treatment with growth hormone-releasing hormone (GHRH) increases brain levels of NAAG and GABA, but not glutamate levels, in the DLPFC in older adults with mild cognitive impairment and cognitively intact older adults (Friedman et al., 2013). Research suggests that a missense mutation in the FOLH1 gene (rs202676 G allele) is linked to reduced levels of NAAG in both unaffected individuals and schizophrenia patients. Additionally, it has been observed that NAAG levels positively associated with visual memory performance, indicating that higher NAAG levels are linked to better cognition, and implying that augmenting NAAG may have the potential to enhance cognitive function (Zink et al., 2020). It is clear from previous literature that smoking causes metabolic changes in the frontal regions of the brain, which are also associated with changes in cognitive function (Janes et al., 2016; Durazzo et al., 2016; Li et al., 2018). However, whether the metabolism of NAAG is related to the cognitive behavior of smoking addicts needs further study. In addition, changes in NAAG as part of glutaminergic neurotransmission and NAA as a compound not involved in neurotransmission may mask each other, possibly leading to false conclusions in nicotine addicts about individual concentrations of both (Jessen et al., 2013). The interactions between metabolites require us to be vigilant in interpreting the obtained results. Small differences between patient characteristics may have contributed to the lack of differences in NAAG metabolism levels in our data. In conclusion, although the findings of this study are small, it provides valuable neuroimaging evidence for investigating the mechanisms underlying nicotine addiction, which is essential for future research on innovative pharmacological for withdrawing nicotine addictive behavior.

In addition, studies have shown that increasing the function of the GABAergic nervous system reduces the activity of the reward circuit, while decreasing its function increases the activity level of the reward circuit. On the contrary, Glu and NAAG are excitatory neurotransmitters, which cause changes in the reward circuit and enhance its function when the function of glutaminergic nervous system is improved (Everitt and Robbins, 2005). This change occurs, for example, when the body is exposed to an addictive substance, which binds to GABAergic or glutaminergic neuronal receptors in the brain, stimulating those neurons resulting in less GABA being released or higher Glu and NAAG being released. The inhibition effect of GABAergic neural pathway on dopaminergic neurons is weakened or the excitatory effect of glutaminergic neural pathway on dopaminergic neurons is enhanced, which leads to the upregulation of the reward circuit function, resulting in addictive behavior (Xi and Gardner, 2008). Combined with the findings of our previous research, smokers have relatively elevated levels of Asp in the mPFC of the brain (Yu et al., 2023). Moreover, many studies have confirmed the imbalance of glutaminergic system in the brain of smokers. At present, our results also confirm the potential positive correlation of NAAG level with nicotine intake and FTND score. Therefore, as a kind of addictive substances, nicotine may stimulate excitatory neurotransmitters (Asp and NAAG), causing the upregulation of the function of the reward circuit, leading to the occurrence of addictive behaviors. Of course, further research is needed to confirm our suspicions.

In addition, the changes in brain metabolites are influenced by multiple factors, such as alcohol consumption, dietary habits, sleep, exercise, and psychological stress. Smokers often have confounding factors such as alcohol consumption, caffeine intake, or chronic stress, which may independently regulate the metabolism of GABA, Glx, and NAAG. Research data suggest that smoking may complicate the study of GABA in the cortical system in alcoholism, and continuous smoking may promote alcohol sobriety (Gulliver et al., 2000); compared with non-alcoholic controls, there was no significant defect in GABA in the occipital cortex 1 week after alcohol abstinence, but only non-smoking alcoholics further decreased 1 month after abstinence. Additionally, in the early stage of alcohol withdrawal, it was found that the absolute concentration of GABA in the occipital cortex of non-smoking patients was higher than that of smoking patients, suggesting that smoking may affect the role of GABA in alcohol dependence and withdrawal (Mason et al., 2006). In 2015, Chitty et al. (2015) found that an increase in the absolute concentration of GSH in patients with bipolar disorder (BD) was associated with a reduction in alcohol consumption and smoking frequency. Dietary components may affect the metabolism of GABA by regulating the cofactors related to GABA synthesis. Additionally, vitamin B6, as a key cofactor of GAD enzyme, insufficient intake may limit GABA production (Kennedy, 2016). A diet rich in polyphenols (such as theanine in green tea) or fermented foods (such as kimchi and yogurt) can promote GABA secretion in the gut or increase its permeability through the blood-brain barrier, thereby altering peripheral and central GABA levels (Strandwitz, 2018). Regular exercise has been shown to increase GABA concentration in the prefrontal cortex, and the mechanism may be related to enhanced neuroplasticity mediated by brain-derived neurotrophic factor (Maddock et al., 2016). As a regulatory peptide of the glutamate system, the level of NAAG may be affected by both the acute and chronic phases of smoking. For example, a single smoking may temporarily increase NAAG by activating nAChR, while the neural adaptability induced by long-term smoking may counteract this effect, resulting in insignificant differences between groups and intra-individual dynamic changes related to smoking behavior. The correlation between NAAG and smoking behavior may be statistically significant but with a small effect size, reflecting that smoking only explains a very small part of the NAAG variation. Its biological significance still requires further longitudinal analysis and verification. Besides. Long-term smoking may trigger compensatory regulation of other neurotransmitters, indirectly masking the population changes of NAAG. Its dynamic fluctuations may still be related to individual smoking behavior. In conclusion, the dynamic changes of metabolites are the result of the combined effects of genetic, environmental and behavioral factors. To enhance the reliability of the research conclusions, future studies need to adopt multivariate models to control the above-mentioned confounding factors and analyze the independent effects and interactions of different lifestyle factors through longitudinal studies.

Despite the important roles of NAAG in psychiatry, particularly in schizophrenia, have explored by Tsai et al. (1995) and Bergeron et al. (2005), the NAAG detection by MRS for clinical studies are still limited, which could be due to a combination of technical, biological, and practical hurdles. Firstly, NAAG is present in the brain at a concentration (0.6–3 mM) roughly 10 times lower than its cousin, NAA (Govindaraju et al., 2000). This makes its signal inherently weak and difficult to be detected with a high SNR, especially in a clinically feasible scan time. Secondly, the resonance peaks of NAAG and the much more abundant NAA overlap significantly in a standard MR spectrum. Therefore, the *J*-editing techniques (e.g., MEGA-PRESS used in this study; Edden et al., 2007) was introduced specifically to tease them apart. However, the *J*-editing method require additional radio-frequency (RF) pulses to selectively and precisely edit the NAAG signal, which makes it highly susceptible to B0 inhomogeneity and B1 imperfections and places high requirement on careful shimming, pulse power calibration, and specialized post-processing software (often developed in-house by research labs; Choi et al., 2010). Future studies may benefit from the higher magnetic fields (7T and above) for increased SNR and spectral resolution and automated processing pipelines for removing expert analysis barrier. Besides, while NAA is a well-established “neuronal health marker,” the precise biological function of NAAG is less understood. Although alterations in NAAG levels have been reported in a wide range of disorders (schizophrenia, epilepsy, ALS, etc.), the changes are often inconsistent and not specific to any single disease (Agarwal and Renshaw, 2012). This lack of diagnostic specificity limits its utility as a standalone clinical tool. The promising near-term application of NAAG detection may lie in the pharmaceutical clinical trials. NAAG is linked to glutamatergic dysfunction, and drugs targeting this system (e.g., for schizophrenia) could use *J*-editing NAAG MRS as a biomarker to prove that the drug is engaging its intended target in the brain.

## 5 Limitations

The inconsistency in ^1^H-MRS results may be related to many factors. Firstly, the selection criteria for research subjects are different (severity of symptoms, duration of abstinence, previous treatment history, and comorbidities, etc.). The current study on smoking addiction was carried out with a relatively small sample size and only with male subjects, therefore, the results cannot be extrapolated to women and sex-differences remain an open question. The statistical analyses in this study were still with limited statistical power (Supplementary Table 4), and further research based on a larger substance addiction database, covering multiple clinical centers, is expected for better understand the mechanisms of nicotine addiction. Secondly, since factors, such as the alcohol, caffeine, diet, sleep, and stress, may influence human brain metabolite levels, future studies are expected with strictly control of these factors. Thirdly, there are many factors that affect the spectral quality of MRS, including the field strength, the quality of field uniformity, the choice of target brain region, voxel size, and spectral analysis algorithms, as a result, many studies have not reached consistent conclusions. The standardized data collection and reconstruction protocols are expected in future studies to promote clinical cohort studies of MRS. Finally, the combination of MRS with brain structural and functional MRI techniques is expected to further study the correlation between changes in brain metabolism and in brain structures or functions.

## 6 Conclusion

This study used *J*-edited ^1^H-MRS to quantitatively detect the levels of metabolites (GABA+, Glx, and NAAG) in the mPFC of smokers and non-smokers, and found that the NAAG level shown potential positive correlation with the FTND score and daily cigarette consumption in smokers. These indicate that smoking may linked to the dysfunction of glutamatergic system in the mPFC of the brain. The NAAG variation may serve as potential biomarker prediction of within-smoker severity.

## Data Availability

The datasets generated during this study are fully available within the article and supplementary materials. If the original data materials are required, please contact the author to obtain them.
