# Supplementary material for: Correlation of N-acetylaspartyl glutamate level in the medial prefrontal cortex with FTND and daily smoking amounts in adult cigarette smokers
Source: Front Neurosci. 2025 Oct 6;19:1647427. doi: 10.3389/fnins.2025.1647427 (PMC12536034; doi:10.3389/fnins.2025.1647427)
Supplement: Supplementary file 1 [file Data_Sheet_1.pdf]

Supplementary Table 1. MRSinMRS checklist for the data acquisition and post-processing of the *J*-edited MRS sequences.

**Site (name or number)**
**1. Hardware**
a. Field strength: 3.0 T.
b. Manufacturer: Philips Healthcare.
c. Model (software version if available): Phillips Ingenia CX 3.0 T.
d. RF coils: 32 channel ^{1}H head coil.
**2. Acquisition**
a. Pulse sequence: MEshcher-GArwood Point Resolved Spectroscopy (MEGA-PRESS).
b. Volume of interest (VOI) locations: Medial prefrontal cortex (mPFC).
c. Nominal VOI size: 30×30×30 mm^3.
d. Repetition time (TR), echo time (TE):<br>GABA/Glx editing: TR = 2000 ms; TE = 68 ms; NAAG editing: TR = 2000 ms; TE = 140 ms.
e. Total number of excitations:<br>GABA/Glx, NAAG: Number of signal averages (NSA) = 192 (both ON and OFF spectra were repeated by 96 times, totally 192 spectra).
f. Additional sequence parameters:<br>Spectral width = 2000 Hz; number of spectral points = 2048; ON/OFF frequency offsets = 1.9/7.5 ppm for GABA editing, 4.61/4.15 ppm for NAAG editing.<br>(spectral width in Hz, number of spectral points, frequency offsets)
g. Water suppression method: VAPOR scheme.
h. Shimming method, reference peak, and thresholds for “acceptance of shim” chosen:<br>FASTMAP shimming of the voxels was performed automatically before each acquisition, yielding water signal line widths of 6 to 10 Hz.
**3. Data analysis methods and outputs**
a. Analysis software: MATLAB; Gannet 3.0; SPM12; Tarquin; SPSS 22.0.
b. Processing steps deviating from quoted reference or product:<br>For the NAAG editing, the edited NAAG signal at 2.60 ppm was fitted using a single Gaussian model. The lower and upper limits of chemical shifts for NAAG signal fitting were 2.5 and 3.0 ppm, respectively.
c. Output measure (eg absolute concentration, institutional units, ratio), processing steps deviating from quoted reference or product:<br>Metabolites with institutional units and metabolite ratios.
d. Quantification references and assumptions, fitting model assumptions:<br>The Cr signal at 3.0 ppm from the OFF-averaged spectum, and the extra aquired 32-averaged water signal were fitted with a single Lorentz model and a mixed Lorentz-Gaussian Model, respectively, for references.<br>For GABA+ editing, the edited GABA+ signal at 3.02 ppm was fitted using a single Gaussian model, while the Glx signal at 3.75 ppm was fitted using a double Gaussian model. For the NAAG editing, the edited NAAG signal at 2.60 ppm was fitted using a single Gaussian model.

**4. Data quality**
a.Reported variables (SNR, linewidth (with reference peaks))
b.Data exclusion criteria:<br>The metabolite levels were included in further statistical analysis only when the fitting errors of GABA+/Cr and Glx/Cr were less than 15%, while the fitting error of NAAG was accepted if it were less than 25%.
c. Quality measures of postprocessing model fitting (eg CRLB, goodness of fit, SD of residual):<br>Fitting error is reported with standard deviation of the residual signal.
d. Sample spectrum

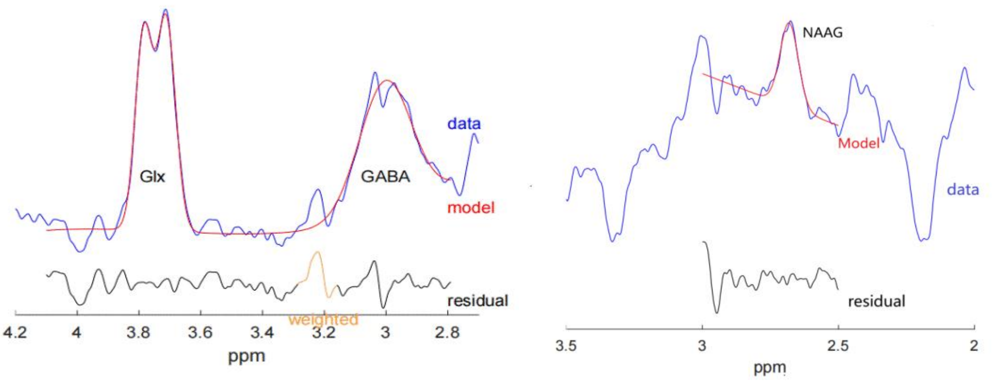

GABA&Glx edited spectrum　　NAAG edited spectrum

Supplementary Table 2:

	GABA+/Cr			Glx/Cr			NAAG/Cr		
	r	P	Adjusted P	r	P	Adjusted P	r	P	Adjusted P
FTND	0.276	0.301	0.452	0.149	0.581	0.792	0.535	0.033*	0.099
RRSQ	0.297	0.265	0.452	0.365	0.164	0.492	0.437	0.090	0.135
BIS-11	-0.061	0.824	0.824	0.067	0.806	0.806	-0.075	0.783	0.783
Initial age of smoking	-0.123	0.649	0.779	0.197	0.463	0.792	-0.456	0.076	0.135
Daily smoking amounts	0.489	0.055	0.330	0.119	0.660	0.792	0.647	0.007*	0.042*
Years of nicotine use	-0.359	0.179	0.452	-0.451	0.080	0.480	0.215	0.425	0.510

	GABA+/water			Glx/water			NAAG/water		
	r	P	Adjusted P	r	P	Adjusted P	r	P	Adjusted P
FTND	0.213	0.429	0.530	0.054	0.843	0.939	0.434	0.093	0.163
RRSQ	0.289	0.278	0.530	0.021	0.939	0.939	0.509	0.086	0.163
BIS-11	-0.216	0.422	0.530	0.126	0.643	0.939	-0.037	0.892	0.892
Initial age of	0.246	0.358	0.530	-0.110	0.685	0.939	-0.416	0.109	0.163

smoking									
Daily smoking amounts	0.170	0.530	0.530	-0.109	0.688	0.939	0.644	0.007*	0.042*
Years of nicotine use	-0.201	0.456	0.530	0.028	0.918	0.939	0.286	0.283	0.340

Supplementary Table 3. The Post-hoc power analysis for the metabolite comparison between smoker and control groups.

Metric	Group Comparison	Detectable effect sizes (α=0.05, two- sided, statistical power > 0.8)	Measured differences
**GABA+/Cr**	Smokers vs Non- smokers	0.019	0.006
**Glx/Cr**	Smokers vs Non- smokers	0.091	0.017
**NAAG/Cr**	Smokers vs Non- smokers	0.010	0.007
**GABA+/Water (i.u.)**	Smokers vs Non- smokers	0.380	0.106
**Glx/Water (i.u.)**	Smokers vs Non- smokers	1.400	0.051
**NAAG/Water (i.u.)**	Smokers vs Non- smokers	0.12	0.091

Supplement Table 4:

The Post-hoc power analysis for the metabolite comparison between smoker and control groups.

Metric	Group Comparison	Achieved Power (α=0.05, two- sided)
**GABA+/Cr**	Smokers vs Non- smokers	0.090
**Glx/Cr**	Smokers vs Non- smokers	0.063
**NAAG/Cr**	Smokers vs Non- smokers	0.375
**GABA+/Water**	Smokers vs Non- smokers	0.112
**Glx/Water**	Smokers vs Non- smokers	0.051
**NAAG/Water**	Smokers vs Non- smokers	0.413

The power was computed using PASS 2023, version 23.0.2.

Supplement Figure 1:

Example fits of the worst-case scenario of the acquired editting spectra for GABA/Glx(＜15%) and NAAG(＜25%)

GABA/Glx:

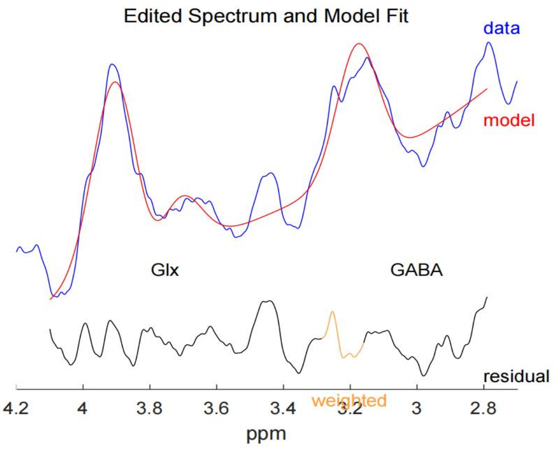

FitErr(GABA) = 14%; FitErr(Glx) = 2.3%

NAAG :

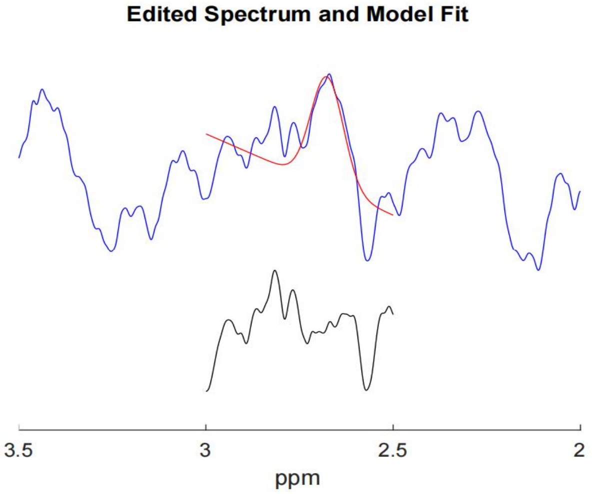

FitErr(NAAG): 24.1%
